# DEVELOPMENT OF THE LONGITUDINAL STUDY OF HEALTH AND AGEING IN KENYA (LOSHAK)

**DOI:** 10.1093/geroni/igad104.3706

**Published:** 2023-12-21

**Authors:** Niranjani Nagarajan, Joshua Ehrlich, Anthony Ngugi, Eunice Mwangi, Roselyter Riang’a, Shaheen Sayed, Muthoni Gichu, Kenneth Langa

**Affiliations:** University of Michigan Kellogg Eye Center, Ann Arbor, Michigan, United States; University or Michigan, Ann Arbor, Michigan, United States; Aga Khan University, Nairobi, Nairobi Area, Kenya; Aga Khan University, Nairobi, Nairobi Area, Kenya; Aga Khan University, Nairobi, Nairobi Area, Kenya; Aga Khan University, Nairobi, Nairobi Area, Kenya; Ministry of Health, Nairobi, Nairobi Area, Kenya; University or Michigan, Ann Arbor, Michigan, United States

## Abstract

In Kenya, the number of adults aged ≥60 is expected to nearly quadruple by 2050, making it one of the most rapidly aging countries in Sub-Saharan Africa (SSA). Accordingly, we designed the Longitudinal Study of Health and Ageing in Kenya (LOSHAK) to generate novel data to address the health and economic consequences of this demographic transition. Modeled on the U.S. Health and Retirement Study (HRS), LOSHAK joins a network of harmonized studies on aging in >45 countries worldwide; however, LOSHAK will be only the second such study in SSA. LOSHAK will advance population aging research in low- and middle-income countries through the study of: (1) biomarkers and physiological measures; (2) the impacts of air pollution and climate vulnerability; (3) Alzheimer’s disease and related dementias, mental health, disability, caregiving, and psychosocial wellbeing; and (4) economic security, including the impact of social welfare. The current feasibility and initial pilot testing phase of LOSHAK is nested within the Kaloleni/Rabai Community Health and Demographic Surveillance System on the coast of Kenya and aims to validate measures and data collection procedures in a purposive sample of Kenyan adults aged ≥ 45 years. Among 205 participants surveyed, the mean age was 64 years, 58% were female, and 37% were currently employed. In the future, a full-scale Wave 1 LOSHAK will collect data from a nationally representative panel of Kenyans aged ≥45 years and will inform future public health and economic policy to address challenges related to rapid aging in Kenya and throughout SSA.

